# Prognostic implication of PTPRH hypomethylation in non-small cell lung cancer

**DOI:** 10.3892/or.2015.4082

**Published:** 2015-06-25

**Authors:** TAKASHI SATO, KENZO SOEJIMA, ERI ARAI, JUNKO HAMAMOTO, HIROYUKI YASUDA, DAISUKE ARAI, KOTA ISHIOKA, KEIKO OHGINO, KATSUHIKO NAOKI, TAKASHI KOHNO, KOJI TSUTA, SHUN-ICHI WATANABE, YAE KANAI, TOMOKO BETSUYAKU

**Affiliations:** 1 Division of Pulmonary Medicine, Department of Internal Medicine, Keio University School of Medicine, Tokyo 160-8582, Japan; 2 Division of Molecular Pathology, National Cancer Center Hospital, Tokyo 104-0045, Japan; 3 Division of Genome Biology, National Cancer Center Research Institute, Tokyo 104-0045, Japan; 4 Department of Pathology and Clinical Laboratories, Pathology Division, National Cancer Center Hospital, Tokyo 104-0045, Japan; 5 Department of Thoracic Oncology, Thoracic Surgery Division, National Cancer Center Hospital, Tokyo 104-0045, Japan

**Keywords:** non-small cell lung cancer, PTPRH, DNA methylation, hypomethylation, prognostic factor

## Abstract

PTPRH is a receptor-type protein tyrosine phosphatase thought to be a potential regulator of tumorigenesis. The aim of the present study was to clarify the significance of *PTPRH* expression and its regulation by DNA methylation in non-small cell lung cancer (NSCLC), especially in lung adenocarcinoma (LADC). *PTPRH* mRNA expression was examined in 89 NSCLC and corresponding non-cancerous tissues. The correlation between DNA methylation and *PTPRH* gene expression was investigated in another cohort that consisted of 145 patients with LADC, a major NSCLC subtype. Gene regulation by DNA methylation was assessed using a DNA methylation inhibitor. *PTPRH* mRNA expression was significantly upregulated in NSCLC. *PTPRH* DNA methylation was reduced in LADC samples and inversely correlated with mRNA expression. 5-Aza-2′-deoxycytidine treatment of lung cancer cell lines with low *PTPRH* expression, restored mRNA *PTPRH* expression levels. Furthermore, low *PTPRH* methylation was associated with shorter recurrence-free survival (P=1.64×10^−4^) and overall survival (P=5.54×10^−5^). Multivariate analysis revealed that *PTPRH* DNA methylation was an independent prognostic factor (P=6.88×10^−3^). It was confirmed that *PTPRH* is overexpressed in NSCLC. Furthermore, we determined that *PTPRH* is epigenetically regulated by DNA hypomethylation, with prognostic implications for LADC.

## Introduction

Lung cancer is the leading cause of cancer-related mortality worldwide (1). Non-small cell lung cancer (NSCLC) accounts for ~85% of all lung malignancies and adenocarcinoma is the most common histological subtype. Although recent progress in diagnosis and treatment including molecular-targeted therapy have provided a considerable survival benefit, ~40% of NSCLC are diagnosed at an advanced stage, with an overall 5-year survival of ~15% and recurrence rates remain high (2). Therefore, further identification of key molecular alterations in NSCLC is required.

Tyrosine phosphorylation is an important signaling mechanism in the regulation of various biological processes. Activation of protein tyrosine kinase (PTK) is a common feature of cancer and many drugs targeting PTKs, such as epidermal growth factor receptor (EGFR)-tyrosine kinase inhibitors, have been introduced. Protein tyrosine phosphatases (PTPs) also regulate tyrosine phosphorylation and are involved in cancer. Recent evidence has shown the relevance of PTPs either as tumor suppressors or oncoproteins (3). Epigenetic and genetic alterations in genes coding PTPs may be associated with cancer phenotypes.

PTPRH, also known as stomach cancer-associated PTP-1 (SAP-1), was first identified as a transmembrane-type PTP abundant in a subset of pancreatic and colorectal cancer cell lines (4). This enzyme belongs to the R3 subtype receptor-type PTP, together with PTPRB (also known as VE-PTP), PTPRJ (also known as DEP-1) and PTPRO and localizes to the microvilli of the brush border in the gastrointestinal tract (5). Ablation of *PTPRH* inhibits tumorigenesis in mice heterozygous for an adenomatous polyposis coli mutation (*Apc*^min/+^) (6). Although it has been suggested that *PTPRH* regulates intestinal tumorigenesis, the mechanism is unclear. In contrast, *PTPRH* was found to be downregulated in advanced human hepatocellular carcinoma (7) and inhibited the proliferation of cultured cells (8,9). Thus, the role of *PTPRH* is largely unknown and needs to be clarified in diseases including lung cancer.

Epigenetic changes such as aberrant DNA methylation in human cancers have been described (10). DNA hypermethylation in the 5′-UTR CpG-rich regions can block the expression of tumor suppressor genes. In NSCLC, silencing of specific genes such as *RASSF1A*, *CDKN2A*, *RARβ*, *MGMT*, *APC*, *DAPK*, *FHIT*, and *CDH13* due to DNA hypermethylation around their promoter regions has been frequently observed (11). However, the role of DNA hypomethylation in permitting overexpression of specific genes is relatively poorly understood in NSCLC.

In the present study, we investigated the expression status of *PTPRH* in NSCLC. Next, it was elucidated that overexpression of *PTPRH* was caused by an underlying mechanism involving DNA hypomethylation. Then, the regulatory mechanism was confirmed *in vitro* using a DNA methylation inhibitor. Furthermore, it was determined that *PTPRH* DNA methylation was an independent prognostic factor for NSCLC patients.

## Materials and methods

### Patients and tissue samples

Two independent cohorts of lung cancer patients were investigated. The first cohort (LC-C1) comprised 89 paired samples of tumorous lung and corresponding non-cancerous tissues from patients with primary NSCLC who underwent lung resection at the Department of Thoracic Surgery, Keio University Hospital, Japan, between 2001 and 2006. Approval for institutional review of these samples was obtained in accordance with the requirements of the Keio University Institutional Review Board (IRB #16-90-1). The second cohort (LC-C2) consisted of 145 paired samples of tumorous lung and corresponding non-cancerous tissues from patients with primary lung adenocarcinoma (LADC) who underwent lung resection at the National Cancer Center Hospital, Japan, between 1997 and 2008. These tissue specimens were provided by the National Cancer Center Biobank, Japan and the present study was also approved by the Ethics Committee of the National Cancer Center, Japan. All patients in the present study provided written informed consent. Clinicopathological parameters in both cohorts are summarized in Table I.

### Cell lines

Three human lung cancer cell lines were used: A549 (adenocarcinoma), VMRC-LCD (adenocarcinoma) and EBC-1 (squamous cell carcinoma). A549 cells were purchased from the American Type Culture Collection (ATCC; manassas, VA, USA). VMRC-LCD and EBC-1 cells were purchased from the Health Science Research Resources Bank (Osaka, Japan). All cell lines were cultured according to the supplier's instructions.

### cDNA microarray analysis

GeneChip Human Genome 2.0 array (Affymetrix, Inc., Santa Clara, CA, USA) was used to monitor the expression profiles of the LC-C1 samples. Total RNA was extracted from tumorous tissues and paired non-cancerous lung tissues using TRIzol (Life Technologies, Carlsbad, CA, USA). The labeled cRNA was prepared using standard Affymetrix protocols. The signal intensities of the probe sets were normalized using the Affymetrix Power Tools RMA method implemented using Resolver software (Rosetta Biosoftware, Seattle, WA, USA).

### Quantitative real-time reverse transcription-polymerase chain reaction (qRT-PCR) analysis

Total RNA extracted from 62 tumorous and 17 non-cancerous tissues in LC-C1, 111 tumorous and 96 non-cancerous tissues in LC-C2 and lung cancer cell lines, was reverse-transcribed to cDNA using TaqMan reverse transcription reagents or SuperScript III reverse transcriptase (both from Life Technologies). For qRT-PCR analysis, TaqMan gene expression assays were used for human *PTPRH* (Hs00936195_m1, #4331182) and human glyceraldehyde-3-phosphate dehydrogenase (GAPDH; #4310884E, both from Life Technologies) to normalize input cDNA. Quantitative analysis was performed using Applied Biosystems 7500 Fast-Real Time PCR system (Life Technologies). All assays were performed in triplicate.

### Infinium assay

Genomic DNA was extracted from all LC-C2 tissue samples and lung cancer cell lines using a QIAamp DNA mini kit (Qiagen, Valencia, CA, USA). Bisulfite conversion using an EZ DNA Methylation-Gold kit (Zymo Research, Irvine, CA, USA) was carried out on 500 ng aliquots of DNA. Subsequently, DNA methylation status at 27,578 CpG loci was examined at single-CpG resolution using the Infinium HumanMethylation27 Bead array (Illumina, San Diego, CA, USA). An Evo robot (Tecan, Männedorf, Switzerland) was used for automated sample processing. After whole genome amplification and hybridization, the specifically hybridized DNA was fluorescence-labeled by a single-base extension reaction and detected using a BeadScan reader (Illumina) in accordance with the manufacturer's protocols. The data were then assembled using GenomeStudio methylation software (Illumina). At each CpG site, the ratio of the fluorescence signal was measured using a methylated probe relative to the sum of the methylated and unmethylated probes, such as the β-value, which ranges from 0 to 1 and reflects the methylation level of an individual CpG site. The reliability of DNA methylation levels (β-values) determined by the Infinium assay was verified in our previous studies (12–14).

### DNA methylation analysis with the MassARRAY system

Bisulfite treatment using an EpiTect Bisulfite Kit (Qiagen GmbH, Hilden, Germany) was carried out on 250 ng of genomic DNA extracted from LC-C2 samples, in accordance with the manufacturer's protocol. DNA methylation levels containing the CpG site cg11261264 in the Infinium assay were evaluated quantitatively using the MassARRAY platform (Sequenom, San Diego, CA, USA). This method utilizes base-specific cleavage and matrix-assisted laser desorption/ionization time-of-flight mass spectrometry (MALDI-TOF MS) (15). Specific PCR primers for bisulfite-converted DNA were designed using the EpiDesigner software package (http://www.epidesigner.com; Sequenom). The forward and reverse primers used were 5′-TTGTGTGTTGTTTGAAGTTAGTGTTT-3′ and 5′-CTAAACCTAAAACTCCTAAATCCCC-3′, respectively. A T7-promoter tag (5′-CAGTAATACGACTCACTATAGGGAGAAGGCT-3′) was added to the reverse primer for *in vitro* transcription and a 10-mer tag was added to each forward primer to balance the PCR. To overcome PCR bias in DNA methylation analysis, we optimized the annealing temperature and type of DNA polymerase: 0, 50 and 100% methylated control DNA (EpiTect methylated human control DNA; Qiagen) was used as template to test the linearity of the protocol. HotStar *Taq* DNA polymerase (Qiagen) was used for the PCR. The PCR products were used as a template for *in vitro* transcription and RNase A-mediated cleavage reaction using an EpiTyPER reagent kit (Sequenom). The fragmented samples were dispensed onto a SpectroCHIP array and then detected on a MassARRAY analyzer compact MALDI-TOF MS instrument. The data were visualized using EpiTYPER Analyzer software v1.0 (Sequenom). The DNA methylation level (%) at each CpG site was determined by comparing the signal intensities of methylated and non-methylated templates. Experiments were performed in triplicate for each sample-CpG site and the mean value for the three experiments was used as the DNA methylation level.

### 5-Aza-2′-deoxycytidine (5-aza-dC) treatment

A549, VMRC-LCD and EBC-1 cells were seeded at a density of 9×10^5^ cells/15 cm dish on day 0 and then allowed to attach for a 24-h period. Then, 5-aza-dC (Sigma-Aldrich, St. Louis, MO, USA) was added to a final concentration of 5 *µ*M. Cells were passaged at a subculture ratio of 1:2 on day 3. 5-Aza-dC was added again to the same final concentration 24 h after replating. Since toxicity was observed during preliminary experiments, the final concentration of 5-aza-dC was reduced to 0.5 *µ*M for EBC-1 cells. Genomic DNA and total RNA were extracted from all cells on days 3 and 6.

### Statistical analysis

Differences in mRNA expression or DNA methylation levels between cancerous and non-cancerous tissues were investigated using the Wilcoxon signed-rank or Mann-Whitney U test, respectively. Correlation between *PTPRH* mRNA expression levels obtained by microarray and qRT-PCR, as well as correlation between DNA methylation and mRNA expression levels of *PTPRH*, were examined using Spearman's correlation test. DNA methylation levels obtained by the MassARRAY system were compared with those by the Infinium assay using the Pearson's correlation test. The correlation between mRNA expression or DNA methylation levels of *PTPRH* and clinicopathological factors was examined using Spearman's correlation, Mann-Whitney U and Kruskal-Wallis test. Survival curves for patients with LADC were analyzed by the Kaplan-Meier method and log-rank test. The *PTPRH* DNA methylation cutoff level was determined in order to maximize the sensitivity and specificity for recurrence by receiver operating characteristic (ROC) curve analysis (16). Multivariate analysis of the influence of variables on recurrence-free survival was performed using the Cox proportional hazards model. Statistical analyses were performed using SPSS 20.0 (SPSS, IBM, Chicago, IL, USA) and GraphPad Prism 5.0 software (GraphPad Software, La Jolla, CA, USA). All P-values were two-sided and P<0.05 was considered statistically significant.

## Results

### PTPRH mRNA expression is increased in NSCLC

**Figure 1:**
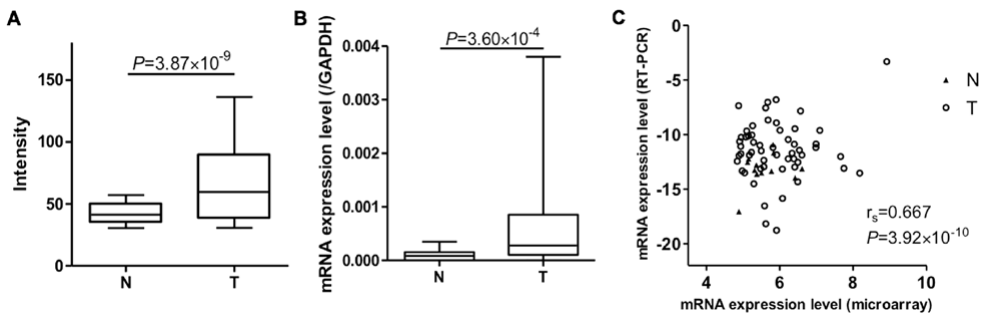
*PTPRH* is significantly upregulated in NSCLC. (A) Microarray gene expression for *PTPRH* in samples of N and of T in LC-C1. *PTPRH* is significantly upregulated in T samples. (B) mRNA expression levels of *PTPRH* in N and the corresponding T samples in LC-C1 obtained by qRT-PCR. *PTPRH* expression is also significantly higher in T samples. (c) Correlation of *PTPRH* mRNA expression between microarray (X-axis; normalized intensity on a log_2_ scale) and qRT-PCR (Y-axis; relative PTPRH/GAPDH expression on a log_2_ scale) analysis for LC-C1 samples. The variables are well correlated. NSCLC, non-small cell lung cancer; N, non-cancerous lung tissue; T, corresponding tumorous tissue; qRT-PcR, quantitative real-time reverse transcription-polymerase chain reaction.

*PTPRH* gene expression in LC-C1 was investigated by cDNA micro-array. The *PTPRH* mRNA expression levels were significantly higher in tumorous tissues compared with the corresponding non-cancerous tissues (Fig. 1A). We performed qRT-PCR on *PTPRH* to confirm the microarray data for 62 cancerous and 17 non-cancerous tissues whose samples were still available. Again, the *PTPRH* mRNA expression levels were significantly higher in tumorous tissues compared with non-cancerous tissues (Fig. 1B). The *PTPRH* mRNA expression data correlated well with that determined by qRT-PCR and microarray analysis (r_s_=0.667; P=3.92×10^−10^; Fig. 1C).

The correlation between *PTPRH* gene expression and clinicopathological parameters are summarized in Table II. Tumor-node-metastasis (TNM) classification was performed according to the Union Internationale Contre le Cancer (UICC)-6 staging system for NSCLC. Distribution of age, gender, histological type, pathological TNM stage, *EGFR* and *K-ras* mutation status except for smoking status did not significantly correlate with *PTPRH* mRNA expression.

### PTPRH DNA methylation is reduced and inversely correlated with mRNA expression in LADC

**Figure 2:**
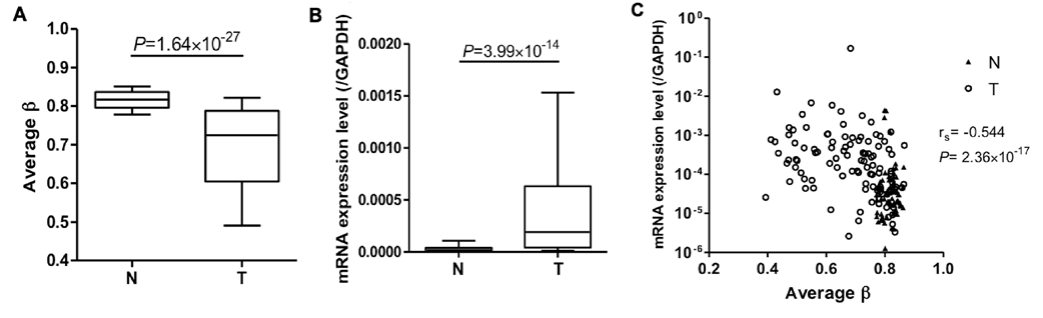
Correlation between DNA methylation and mRNA expression levels for PTPRH. DNA methylation (average values) (A) and mRNA expression levels (B) for PTPRH in samples of N and of T in LC-C2 were determined by the Infinium assay and qRT-PCR analysis, respectively. DNA methylation levels for PTPRH were significantly lower in T than in N samples and levels of PTPRH mRNA expression were significantly higher in T than in N samples. (C) Correlation of DNA methylation (average values) and mRNA expression levels for PTPRH in LC-C2 samples. PTPRH mRNA expression levels were inversely correlated with DNA methylation of the single CpG site. These results suggested that PTPRH DNA hypomethylation may result in increased mRNA expression in tissue samples from the same cohort. N, non-cancerous lung tissue; T, corresponding tumorous tissue; qRT-PCR, quantitative real-time reverse transcription-polymerase chain reaction.

Based on the hypothesis that altered mRNA expression may be caused by aberrant DNA methylation, we investigated the DNA methylation status of *PTPRH* in LC-C2 samples for which genome-wide DNA methylation profiles were available. As there was no significant correlation between *PTPRH* mRNA expression levels and histological type, we used LC-C2 samples and focused on LADC, a major histological type in LC-C1, for further analysis of the epigenetic regulation of *PTPRH*. DNA methylation levels of the CpG site cg11261264, located in *PTPRH* intron 1, were significantly decreased in tumorous compared with non-cancerous tissues (Fig. 2A). We also examined *PTPRH* mRNA expression levels in LC-C2 samples by qRT-PCR. This showed that *PTPRH* mRNA expression levels were again higher in tumorous tissues compared with the corresponding non-cancerous (Fig. 2B) and inversely correlated with DNA methylation of this single CpG site (Fig. 2C). These data suggested that *PTPRH* DNA hypomethylation may result in increased mRNA expression in tissue samples from the same cohort.

### PTPRH gene expression is regulated by DNA hypomethylation

**Figure 3:**
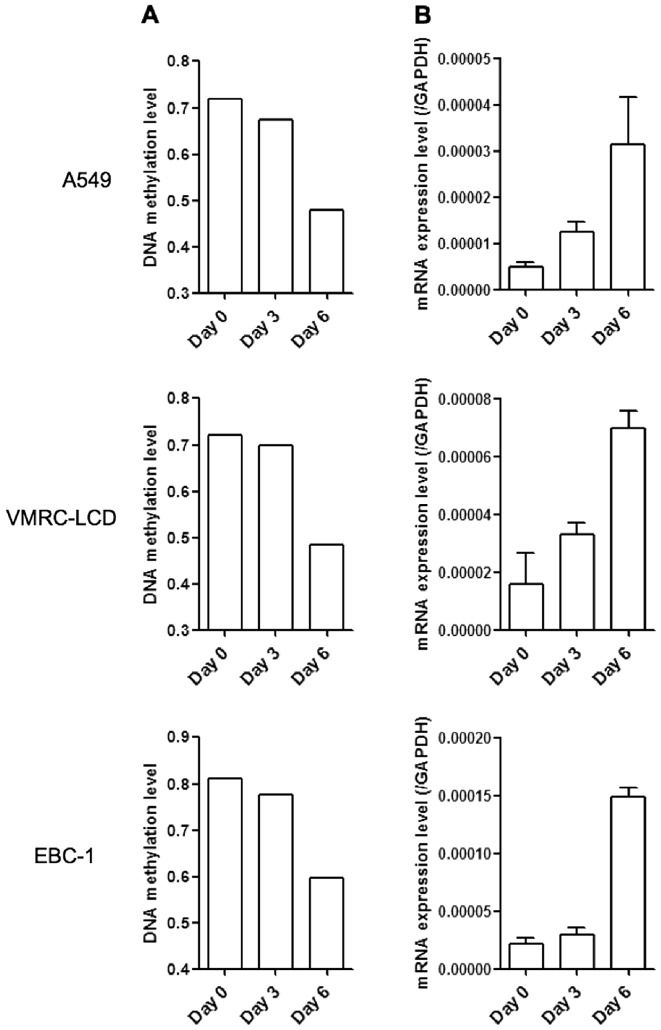
DNA methylation and mRNA expression levels after 5-aza-2′-deoxycytidine (5-aza-dC) treatment. (A) DNA methylation (average values) and (B) mRNA expression levels for *PTPRH* were determined by the Infinium assay and qRT-PCR analysis, respectively. The error bars represent the standard deviation for triplicate qRT-PCR analyses. DNA methylation and mRNA expression levels on days 3 and 6 were compared with those of untreated cells. After 5-aza-dC treatment, reduction of DNA methylation levels and restoration of the *PTPRH* mRNA expression levels were observed in both of the cell lines used. qRT-PCR, quantitative real-time reverse transcription-polymerase chain reaction.

To improve understanding of the influence of DNA methylation on *PTPRH* gene expression, lung cancer cell lines were treated with the DNA methylation inhibitor, 5-aza-dC. In three lung cancer cell lines A549, VMRC-LCD, and EBC-1 with low *PTPRH* mRNA expression, 5-aza-dC treatment induced a marked reduction of DNA methylation and restored *PTPRH* mRNA expression levels (Fig. 3). These data suggested that increased expression of PTPRH was primarily regulated by DNA hypomethylation in LADC.

### DNA hypomethylation of PTPRH as a prognostic factor

**Figure 4:**
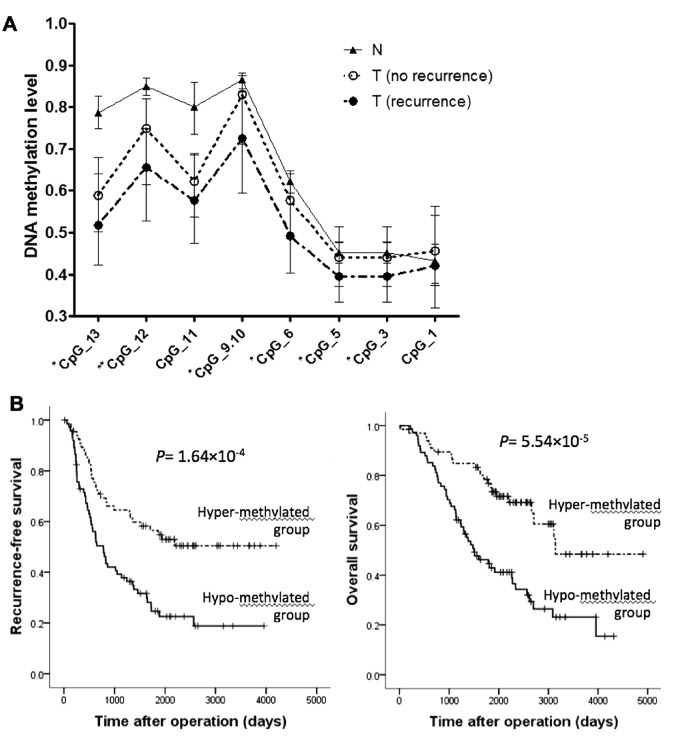
(A) Median DNA methylation levels of *PTPRH* in N and T with no recurrence and those with recurrence in LC-C2. DNA methylation levels of each CpG unit were evaluated quantitatively using the MassARRAY system. The error bars are defined by 25/75% quartiles. DNA methylation levels at most CpG sites showed a significant decrease in tumorous tissue with recurrence compared with those with no recurrence. Significantly different methylated CpG sites are marked as ^*^P<0.05 and ^**^P<0.01). (B) Kaplan-Meier survival curves of patients with *PTPRH* hypermethylation (>0.782 at CpG_9.10) and hypomethylation (≤0.782). The cutoff was determined by ROC curve analysis. The recurrence-free (P=1.64×10^−4^) and overall (P=5.54×10^−5^) survival rates of patients with hypomethylation were significantly lower compared to those of patients with hypermethylation (log-rank test). N, non-cancerous lung tissue; T, corresponding tumorous tissue; ROC, receiver operating characteristic.

Because investigation of DNA methylation by the MassARRAY system allowed a comprehensive coverage of CpG sites, we assessed DNA methylation levels at the relatively CpG-rich region containing the CpG site cg11261264 in the same LC-C2 samples using the MassARRAY system (Genomic positions of the CpG sites are shown in Table III). DNA methylation levels obtained by the MassARRAY system and Infinium assay correlated well (r=0.952, P=1.44×10^−73^), confirming the reliability of the latter assay. DNA methylation patterns quantified at each analyzed CpG site in non-cancerous, tumorous tissue with no recurrence and those with recurrence are shown in Fig. 4A. The consecutive CpG sites CpG_9.10 provided one measured value as a 'CpG unit̓ by the massARRAy system. DNA methylation levels at most CpG sites showed a significant decrease in tumorous tissues with recurrence compared to those with no recurrence. The correlation between the DNA methylation levels of cancerous tissues at CpG_9.10 and clinicopathological parameters are summarized in Table IV. DNA hypomethylation at this CpG unit was significantly correlated with male gender, heavy smoking status, advanced pathological stage and wild-type EGFR.

Kaplan-Meier analysis based on the optimal cutoff determined by ROC curve analysis showed that patients with hypomethylation at the CpG unit had a shorter recurrence-free and overall survival compared with patients with hypermethylation (Fig. 4B; P=1.64×10^−4^ and P=5.54×10^−5^, respectively). Since multiple covariates can affect patient survival, we performed multivariate analysis to confirm that the DNA methylation status of *PTPRH* is an independent prognostic factor for LADC patients. In multivariate Cox proportional hazards regression analysis, *PTPRH* DNA methylation, together with gender and pathological TNM status, emerged as an independent prognostic factor for recurrence-free survival (Table V).

## Discussion

Although some studies have demonstrated the involvement of PTPRH in cancer, especially in intestinal tumorigenesis (4,12,17), its role in lung cancer and the molecular mechanisms underlying its regulation have not been clarified. In the present study, we examined *PTPRH* expression in NSCLC and focused on its regulation by DNA methylation and its clinicopathological implications.

First, we showed that *PTPRH* expression is increased in NSCLC using cDNA microarray analysis. mRNA expression levels obtained from qRT-PCR confirmed the microarray data. While *PTPRH* expression was found to be increased in human colon and pancreatic cancer (4,17) and reduced in advanced human hepatocellular carcinoma (7), to our knowledge no previous study has examined its expression in lung cancer. Our data from LC-C1 samples suggested that *PTPRH* may have a significant role in NSCLC and be associated with its clinicopathological features. In fact, *PTPRH* expression was associated with smoking status that is a well-known risk factor for NSCLC. However, no other association was observed at a statistically significant level.

As *PTPRH* was upregulated in NSCLC, we became interested in the molecular mechanisms underlying its increased expression. Recent studies analyzed the genome-wide DNA methylation profiles of LADC (13,14). We investigated the DNA methylation levels of LC-C2 samples using data from the Infinium assay. As LC-C2 consists of patients with LADC only, we focused on *PTPRH* in LADC, a major subtype of NSCLC, to concentrate on the correlation between measured values and clinicopathological parameters and patient survival. This showed that *PTPRH* DNA methylation was reduced in LADC and inversely correlated with mRNA expression. Several studies identified tumor-specific hypermethylation of other types of PTPs, such as *PTPRO* (18,19), *PTPRD* (20), *PTPRG* (21,22), *PTPN6* (23–25) and *PTPN13* (26), reviewed by Jacob and Motiwala (27), and Julien *et al* (3). However, tumor-specific hypomethylation of PTP has not been reported. 5-aza-dC treatment of human lung cancer cell lines with low *PTPRH* expression restored *PTPRH* mRNA expression levels indicating that *PTPRH* is reactivated by DNA hypomethylation during lung tumorigenesis.

We speculated that PTPRH may play an oncogenic role in lung tumorigenesis because PTPRH is thought to promote intestinal tumorigenesis (6). We attempted a transient knockdown of PTPRH using siRNA in lung cancer cell lines. However, we were unable to find any lung cancer cell lines where cell proliferation was inhibited by its knockdown (data not shown). Although PTPRH was shown to have a capacity to activate Src (28), Src kinase may not be responsible for its oncogenic properties. Sadakata *et al* reported that ablation of PTPRH did not reduce the levels of c-Src activity in *Apc*^min/+^ mice (6) and we did not observe changes in Src phosphorylation (Y416) in PTPRH-knockdown cells (data not shown). Recently, the substrate specificity of the R3 subtype of receptor-type PTPs toward receptor tyrosine kinases was described (29). Further studies are needed to clarify the mechanisms downstream of PTPRH.

We further confirmed the data from the Infinium assay by using the MassARRAY platform and investigated the clinicopathological and prognostic significance of *PTPRH* DNA methylation in LADC. *PTPRH* DNA hypomethylation was significantly correlated with male gender, heavy smoking status, pathological stage and wild-type EGFR. Seo *et al* described the correlation between *PTPRH* expression and *K-ras* mutations in colorectal cancer (17). However, no correlation was found in the present study, probably due to differences in histology. Although the underlying significance of these results and the precise function of PTPRH were not clarified in the present study, our analysis explored *PTPRH* hypomethylation as a novel prognostic factor. To the best of our knowledge, this is the first study to show that *PTPRH* is regulated by DNA methylation and its hypomethylation is related to poor prognosis in LADC.

In conclusion, *PTPRH* is upregulated in NSCLC and it is regulated by DNA hypomethylation in LADC. Moreover, the DNA methylation status of *PTPRH* has been identified as a novel prognostic factor for LADC. Further studies are warranted to clarify its molecular role in the development of NSCLC.

## Figures and Tables

**Table I:** Clinicopathological parameters of patients with NSCLCs in LC-C1 and LC-C2.

Clinicopathological parameters	LC-C1 (n=89)	LC-C2 (n=145)
Age (years)		
Median	68	61
Interquartile range	60–75	55–66
Gender		
Male	56	81
Female	33	64
Smoking status (pack-year)		
Median	30	13
Interquartile range	0–60	0–41
Histological type		
Adenocarcinoma	54	145
Squamous cell carcinoma	24	0
Large cell carcinoma	6	0
Others	5	0
Tumor size (cm)		
Median	3	2.8
Interquartile range	2.5–4.0	2.2–4.5
Tumor stage		
T1	41	64
T2	30	63
T3-4	18	18
Nodal status		
N0	60	94
N1	14	24
N2-3	15	27
Metastatic status		
M0	87	145
M1	2	0
Pathological TNM stage		
I	50	83
II	12	31
III–IV	27	31
*EGFR* mutation		
Wild-type	63	73
Mutant	26	48
*K-ras* mutation		
Wild-type	84	109
Mutant	5	12

TNM, tumor-node-metastasis.

**Table II:** Correlation between mRNA expression levels (microarray) of *PTPRH* and clinicopathological parameters of patients with non-small cell lung cancers.

Clinicopatho-logical parameters	No.	Median intensity (interquartile range)	P^a^-value
Age (years)			5.94×10^−1^^b^ (r_s_=−0.057)
Gender			
Male	56	63.9 (41.4–95.1)	9.56×10^−2^^c^
Female	33	47.3 (37.7–84.2)	
Smoking status (pack-year)			**6.73×10^−3^**^b^ (r_s_=0.287)
Histological type			
Adenocarcinoma	54	49.9 (35.4–84.9)	1.51×10^−1^^d^
Squamous cell carcinoma	24	62.0 (46.1–91.2)	
Large cell carcinoma	6	84.9 (59.6–114.3)	
Others	5	74.9 (64.0–97.3)	
Tumor stage			
T1	41	49.5 (38.9–85.0)	3.48×10^−1^^b^
T2	30	71.0 (36.7–92.5)	
T3-4	18	60.2 (38.2–105.7)	
Nodal status			
N0	60	60.2 (41.5–93.6)	2.81×10^−1^^b^
N1	14	60.5 (34.7–89.6)	
N2-3	15	47.3 (35.3–87.5)	
Metastatic status			
M0	87	56.8 (37.7–87.5)	7.63×10^−2^^b^
M1	2	119.4 (114.3–124.5)	
Pathological			
TNM stage			
I	50	57.8 (38.9–92.5)	8.72×10^−1^^b^
II	12	62.0 (44.8–83.9)	
III–IV	27	59.9 (35.3–114.3)	
*EGFR* mutation			
Wild-type	63	63.7 (43.8–92.5)	1.56×10^−1^^b^
Mutant	26	46.1 (35.9–84.2)	
*K-ras* mutation			
Wild type	84	58.2 (38.6–89.8)	7.91×10^−1^^b^
Mutant	5	59.9 (30.6–84.9)	

a P-values of <0.05 are bold print.

b Spearman's correlation test.

c Mann-Whitney u-test.

d Kruskal-Wallis test; TNM, tumor-node-metastasis.

**Table III:** CpG sites analyzed by the MassARRAY system.

CpG site	Position^a^
CpG_1	Chromosome 19: 55,720,456
CpG_2 (not covered)	Chromosome 19: 55,720,467
CpG_3	Chromosome 19: 55,720,485
CpG_4 (not covered)	Chromosome 19: 55,720,516
CpG_5	Chromosome 19: 55,720,534
CpG_6	Chromosome 19: 55,720,554
CpG_7 (not covered)	Chromosome 19: 55,720,565
CpG_8 (not covered)	Chromosome 19: 55,720,668
CpG_9.10^b^	Chromosome 19: 55,720,728. 55,720,737
CpG_11	Chromosome 19: 55,720,777
CpG_12	Chromosome 19: 55,720,786
CpG_13	Chromosome 19: 55,720,817

a National Center for Biotechnology Information (NCBI) Database (Genome Build 37).

b CpG site identical to cg11261264 in the Infinium assay.

**Table IV:** Correlation between DNA methylation levels of PTPRH and clinicopathological parameters of patients with lung adenocarcinomas.

Clinicopathological parameters	No.	Median DNA methylation level (interquartile range)	P-value^a^
Years of age			7.01**×**10^−2^^b^ (r_s_=0.153)
Gender			
Male	81	0.723 (0.597–0.830)	**3.05×10^−3^**^c^
Female	64	0.833 (0.690–0.870)	
Smoking status (pack-year)			**3.51×10^−3^**^b^ (r_s_=−0.244)
Tumor stage			
T1	64	0.793 (0.672–0.850)	3.15**×**10^−1^^b^
T2	63	0.727 (0.593–0.850)	
T3–4	18	.0.737 (0.627–0.872)	
Nodal status			
N0	94	0.793 (0.627–0.860)	1.96**×**10^−1^^b^
N1	24	0.768 (0.602–0.0.845)	
N2-3	27	0.713 (0.643–0.797)	
Pathological TNM stage			
I	83	0.807 (0.627–0.867)	**1.57×10^−2^**^b^
II	31	0.767 (0.653–0.853)	
III	31	0.703 (0.600–0.790)	
*EGFR* mutation			
Wild-type	73	0.717 (0.547–0.843)	**7.28×10^−3^**^b^
Mutant	48	0.820 (0.647–0.873)	
*K-ras* mutation			
Wild-type	109	0.767 (0.617–0.857)	9.39**×**10^−2^^b^
Mutant	12	0.665 (0.505–0.755)	

a P-values of <0.05 are in bold print.

b Spearman's correlation test.

c Mann-Whitney U-test, TNM, tumor-node-metastasis.

**Table V:** Multivariate analysis of predictive factors for recurrence-free survival in patients with LADCs (Cox proportional hazard model).

Variables	Multivariate analysis Hazard ratio (95% confidence interval)	
Gender	1.801 (1.012–3.205)	**4.53×10^−2^**
Smoking status (pack-year)	1.000 (1.000–1.001)	1.72×10^−1^
Pathological TNM stage	1.468 (1.276–1.690)	**8.69×10^−8^**
*EGFR* mutation	1.490 (0.923–2.407)	1.03×10^−1^
*PTPRH* DNA methylation	0.134 (0.031–0.576)	**6.88×10^−3^**

Bold, P-values of <0.05. TNM, tumor-node-metastasis.

## Abbreviations

NSCLC: non-small cell lung cancer
PTK: protein tyrosine kinase
EGFR: epidermal growth factor receptor
PTP: protein tyrosine phosphatase
SAP-1: stomach cancer-associated protein tyrosine phosphatase-1
LADC: lung adenocarcinoma
qRT-PcR: quantitative real-time reverse transcription-poly-merase chain reaction
MALDI-TOF MS: matrix-assisted laser desorption/ionization time-of-flight mass spectrometry
5-aza-dC: 5-aza-2′-deoxycytidine
ROC: receiver operating characteristic
TNM: tumor-node-metastasis
UICC: Union Internationale Contre le Cancer
